# Significance of Pulmonary Arterial Pressure as a Prognostic Indicator in Lung-Dominant Connective Tissue Disease

**DOI:** 10.1371/journal.pone.0108339

**Published:** 2014-09-30

**Authors:** Atsushi Suzuki, Hiroyuki Taniguchi, Naohiro Watanabe, Yasuhiro Kondoh, Tomoki Kimura, Kensuke Kataoka, Toshiaki Matsuda, Toshiki Yokoyama, Koji Sakamoto, Osamu Nishiyama, Yoshinori Hasegawa

**Affiliations:** 1 Department of Respiratory Medicine and Allergy, Tosei General Hospital, Seto, Aichi, Japan; 2 Department of Respiratory Medicine, Nagoya University Graduate School of Medicine, Nagoya, Aichi, Japan; 3 Department of Respiratory Medicine and Allergology, Kinki University Faculty of Medicine, Osaka-sayama, Osaka, Japan; Keio University School of Medicine, Japan

## Abstract

**Background:**

Lung-dominant connective tissue disease (LD-CTD) is a new concept for classifying the subset of patients with interstitial pneumonia who have clinical features suggesting an associated CTD, but whose features fall short of a clear diagnosis of CTD under the current rheumatologic classification systems. The impact of mean pulmonary arterial pressure (MPAP) in LD-CTD has not been sufficiently elucidated.

**Objectives:**

To evaluate the survival impact of MPAP measured during the initial evaluation in patients with LD-CTD.

**Methods:**

We retrospectively analyzed the initial evaluation data of 100 LD-CTD patients undergoing pulmonary function test, 6-min walk test (6MWT), and right heart catheterization (RHC).

**Results:**

The mean MPAP was 16.2±4.4 mm Hg, and 18 patients had MPAP≥20 mm Hg. A univariate Cox proportional hazard model showed that MPAP and several variables have a statistically significant impact on survival. With stepwise, multivariate Cox proportional analysis, MPAP (HR  = 1.293; 95% CI 1.130–1.480; p<0.001) and mean forced vital capacity (FVC) % predicted (HR = 0.958; 95% CI 0.930–0.986; p = 0.004) were shown to be independent determinants of survival.

**Conclusions:**

Higher MPAP and lower %FVC at the initial evaluation were significant independent prognostic factors of LD-CTD. MPAP evaluation provides additional information of disease status and will help physicians to predict mortality in LD-CTD.

## Introduction

Pulmonary hypertension (PH) has many causes and is a source of significant mortality in affected individuals. Development of PH in the context of interstitial lung disease (ILD) is a well-recognized complication [1]–[5]. Meanwhile, some studies suggest that connective tissue disease (CTD)-associated PH is a prognostic factor, seen especially in systemic scleroderma (SSc) and mixed connective tissue disease (MCTD) [6]–[9]. In addition, there is accumulating evidence that borderline PH (25> mean pulmonary arterial pressure (MPAP) >20 mmHg) may be clinically relevant in both ILD and CTD [2], [10].

Recently, Fischer et al. [11] proposed “lung-dominant CTD” (LD-CTD) as a new concept for classifying the subset of patients with interstitial pneumonia who have clinical features suggesting an associated CTD, but whose features fall short of a clear diagnosis of CTD under the current rheumatologic classification systems. Little information is available regarding prognostic factors in LD-CTD [12]. Considering the importance of PH in ILD and CTD, we assumed that PH might occur in the clinical course of LD-CTD patients and contribute to poor prognosis. The aim of this study was to evaluate whether MPAP predicts survival in LD-CTD patients in whom background, pulmonary function test, 6-min walk test (6MWT), and right heart catheterization (RHC) could be evaluated at the initial evaluation.

## Methods

### Study subjects

**Figure 1:**
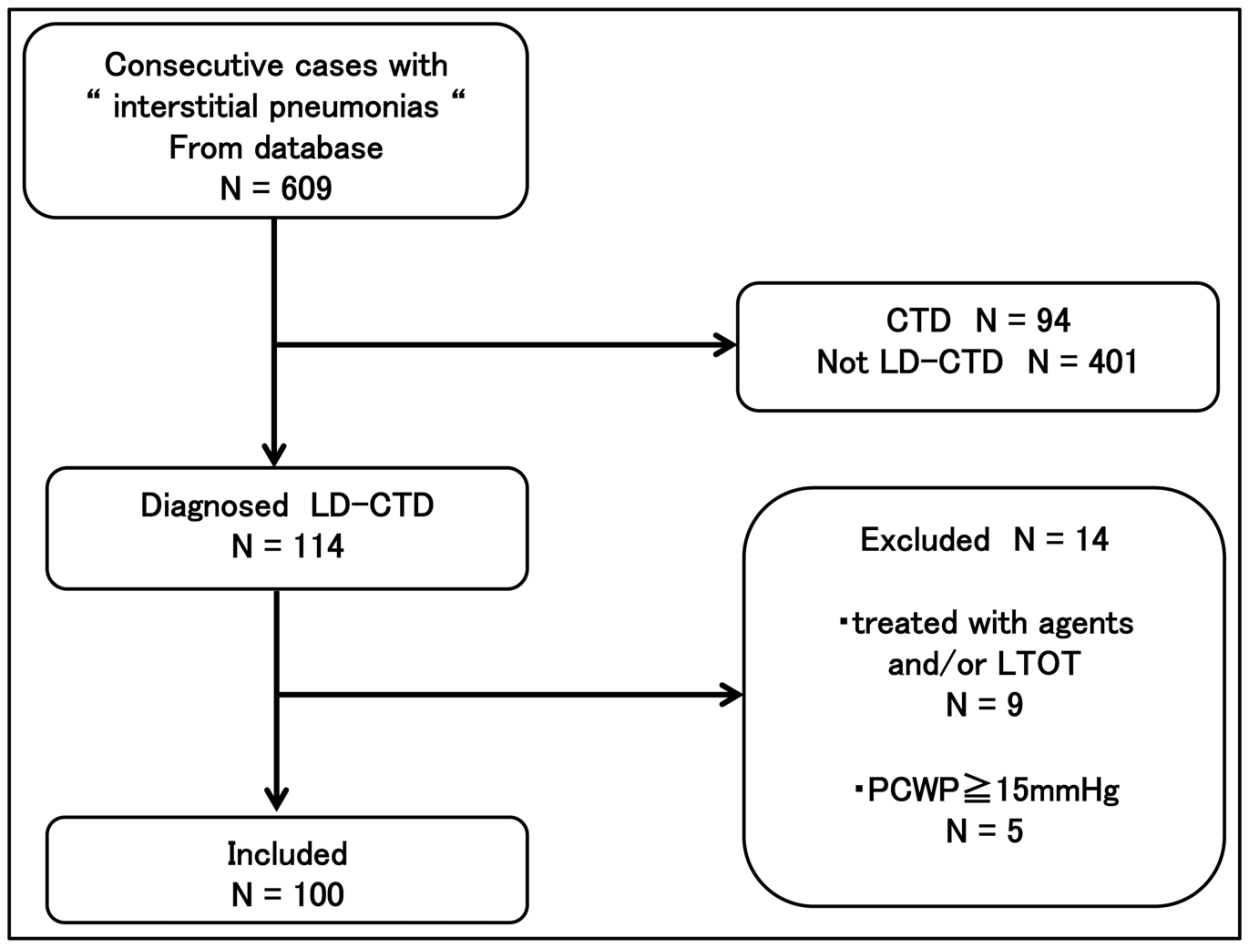
Screening and inclusion process for patients in the study.

Six hundred nine patients with ILD were identified from among patients who underwent systematic evaluations between May 2007 and August 2012 at Tosei General Hospital**.** Systematic evaluations included pulmonary function test, 6MWT, and RHC. LD-CTD was diagnosed based on the criteria proposed by Fischer et al. when specific autoantibodies were present in the absence of extrathoracic features of a definite CTD [11]. Serological tests, including those for antinuclear antibody (ANA), rheumatoid factor (RF), anti-citrullinated peptide (CCP), anti-Scl 70 (Scl-70), anti-Ro/SSA, anti-La/SSB, anti-double-stranded DNA (dsDNA), anti-Smith, anti-ribonucleoprotein (RNP), anti-tRNA synthetase (Jo-1), and anti-centromere antibodies, were performed routinely when ILD was first suspected. Anti-PM-Scl antibodies were unmeasured. Patients were considered to have LD-CTD if they had one of the specific autoantibodies within one year of initial evaluation (Table 1). CTD was diagnosed when patients fulfilled the established criteria for rheumatoid arthritis, systemic lupus erythematosus (SLE), SSc, polymyositis/dermatomyositis, Sjogren’s syndrome (SjS), or MCTD [13]–[18]. Clinically amyopathic dermatomyositis (CADM) was diagnosed when a patient had a skin rash characteristic of DM without clinical evidence of muscle disease and with little or no increase in the serum creatine kinase (CK) level [16], . We excluded any patient with a pre-existing diagnosis of CTD. The following cases were also excluded: (1) patients had been treated with corticosteroids, immunosuppressive agents, PH targeted therapy and oxygen therapy before initial evaluation, (2) RHC was not performed within one month of initial evaluation, or pulmonary artery wedge pressure (PAWP) on RHC was over 15 mm Hg. Finally, 100 patients with LD-CTD were taken as subjects (Figure 1).

**Table 1:** Diagnostic criteria for lung-dominant connective tissue disease.

1. Interstitial pneumonia suggested by high-resolution CT and
2. Insufficient extrathoracic features to allow a specific CTD designation and
3. No identifiable alternative etiology for IP and
4. Any one of the following autoantibodies within one year of initial evaluation
Autoantibodies
a. High-titer ANA (>1∶320) or RF (>60 IU/ml)
b. Nucleolar-ANA
c. Anti-CCP
d. Anti-Scl-70
e. Anti-Ro/SS-A
f. Anti-La/SS-B
g. Anti-ds DNA
h. Anti-Smith
i. Anti-RNP
j. Anti-tRNA synthetase (Jo-1)
k. Anti-centromere

Criteria derived from Reference 11.

ANA: antinuclear antibody; RF: rheumatoid factor; CCP: cyclic citrullinated peptide; Scl-70: Sclero 70; ds DNA: double-stranded DNA; RNP: ribonucleoprotein.

In this study, PH is defined by MPAP≥20 mm Hg (including borderline PH). A previous study indicated that MPAP>20 mm Hg is optimal for predicting the prognosis in IPF patients [2].

This study was approved by the Tosei General Hospital Institutional Review Board (IRB No.398). Patient approval and/or informed consent were waived because the study involved a retrospective review of patient records. Our institutional review board determined that ethical approval was not necessary and did not require the patient’s approval or informed consent.

### Measurements

We studied patients’ characteristics, pulmonary function tests, PaO2, 6MWT, and hemodynamics. All patients underwent spirometry (CHESTAC-55V; Chest, Tokyo, Japan), according to the method described in the American Thoracic Society (ATS) 1994 update [20]. Single-breath diffusing capacity of the lung for carbon monoxide (DLCO) was also measured (CHESTAC-55V). The values for forced vital capacity (FVC) and DLCO were related to % predicted values [21]. 6MWT was conducted according to the ATS statement [22]. Briefly, all patients were tested under standardized conditions by trained technicians. Patients were instructed to walk as far as possible in 6 min, and the distance that patients could walk was recorded [23]. Oxygen saturation was also measured by pulse oximetry at rest for 5 min prior to and immediately after the test. Dyspnea was assessed with the modified Medical Research Council score (MMRC) scale, which includes 5 grades (0–4) of various physiological activities that provoke dyspnea [24]. After the patients had read the descriptive phrases, they selected the number that best corresponded to their level of dyspnea in daily living. Patients underwent Doppler echocardiography. Estimated right ventricular systolic pressure (ERVSP) was measured by the peak tricuspid regurgitant flow velocity using the modified Bernoulli equation [25]–[26]. RHC was performed using a Swan-Ganz catheter percutaneously via either the cubital vein or the femoral vein. At the time of catheterization, the pressure transducers were adjusted to reflect the midthorax in each patient before beginning the procedure. Cardiac output was measured with a thermodilution method. PAWP was measured at the end-expiratory phase.

### Statistical analysis

Survival status was analyzed in February 2014. Continuous variables were expressed as means ± SD. Categorical variables were summarized by frequency. The MMRC score was analyzed as a continuous variable. Distribution of continuous variables was evaluated using the Shapiro-Wilk test. If both variables had a normal distribution, correlations were calculated using Pearson’s correlation test. If either variable had a nonnormal distribution, correlations were calculated using Spearman’s correlation test. When two continuous variables were compared, the t test was used for normal distributions and the Mann-Whitney test was used for nonnormal distributions.Univariate Cox’s proportional hazard models were used to examine the association of selected variables with survival. Variables that were significant (p<0.05) in the univariate analysis were included in the multivariate model. To avoid multicolinearity, only one of the highly correlated variables (coefficient of correlation ≥0.9) was entered in the multivariate model, if present. A stepwise multivariate Cox’s proportional hazards model was then used for variables that were revealed to be significant with the univariate model, in order to select more significant variables. Using the methods of Kaplan-Meier and the log-rank test, we studied the impact on survival of variables. All tests were performed at a significance level of p<0.05. Analysis was completed using IBM SPSS statistics version 19.

## Results

**Figure 2:**
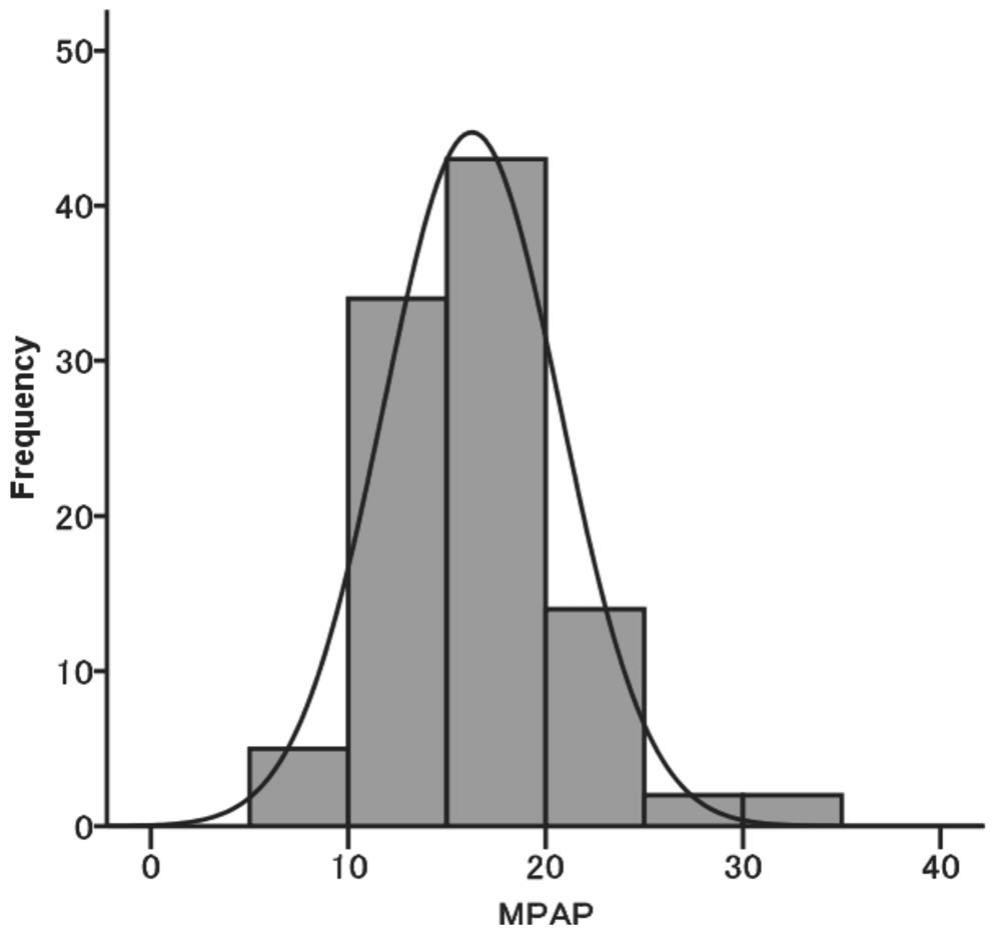
Histogram of MPAP.

The baseline characteristics of 100 patients are summarized in Table 2, and the autoantibody profile of the study cohort is shown in Table 3. ANA was the most frequently positive autoantibody. Seventy-two patients were positive for one antibody, 23 were positive for two antibodies, and 5 had three positive serological tests. Fifty patients underwent surgical lung biopsy. RHC data are summarized in Table 2. MPAP, cardiac index, PAWP and pulmonary vascular resistance (PVR) were 16.2±4.4 mm Hg, 3.10±0.72 lmin^−1^m^−2^, 7.1±3.4 mm Hg, and 1.79±0.96 Wood units, respectively. A histogram of MPAP is shown in Figure 2. Four patients had MPAP>25 mm Hg, and 18 patients had MPAP≥20 mm Hg. In addition, there was a significant but weak correlation between ERVSP by Doppler echocardiography and systolic PAP (r = 0.245; p = 0.021).

**Table 2:** Baseline characteristics.

Variables	Mean	Range
Sex(M/F)	64/36	
Age, years	65.1±8.0	36–79
BMI	23.7±3.9	12.5–33.9
Smoking status		
Ever/never	61/39	
FVC, %predicted	81.1±20.3	30.0–140.0
DLCO, %predicted	56.4±19.1	22.2–107.8
PaO_2_, mmHg	80.8±11.7	55.9–111.0
MMRC	1.0±1.0	0–4
6MWD, m	556.0±143.4	135–1085
Lowest SpO_2_, %	84.4±8.7	52–96
MPAP, mmHg	16.2±4.4	8–31
Cardiac index, lmin^−1^m^−2^	3.10±0.72	0.90–5.71
PAWP, mmHg	7.1±3.4	−2–14
PVR, Wood units	1.79±0.96	0.19–5.51

Data are presented as means ± SD or numbers.

BMI: body mass index; FVC: forced vital capacity; DLCO: diffusing capacity of the lung for carbon monoxide; MMRC: Modified Medical Research Council Dyspnea Scale; 6MWD: 6 minute walk distance; MPAP: mean pulmonary artery pressure; PAWP: pulmonary artery wedge pressure; PVR: pulmonary vascular resistance.

N = 100 except for FVC (n = 99), DLCO (n = 96), MMRC (n = 95), 6MWD (n = 91), Lowest SpO2 (n = 91).

**Table 3:** Autoantibody profile of the study cohort.

Serological tests	N = 100
High-titer ANA (>1∶320) or RF (>60 IU/ml)	57/100
Nucleolar-ANA	26/100
Anti-CCP	14/88
Anti-Scl-70	2/99
Anti-Ro/SS-A	13/100
Anti-La/SS-B	1/93
Anti-ds DNA	5/99
Anti-Smith	0/88
Anti-RNP	1/88
Anti-tRNA synthetase (Jo-1)	2/99
Anti-centromere	6/78

Data are presented as number. Some patients had multiple positive serological tests.

In the follow-up period, six patients showed a definite CTD. Rheumatoid arthritis was diagnosed in two cases, SSc in two, polymyositis in one, and SjS in one using the established criteria [13], [15]–[17].

Fifty-four patients received pharmacologic therapy during the follow-up period. Thirty-nine patients were treated with corticosteroid and immunosuppressive agent, thirty-seven patients were treated with pirfenidone, and five patients were treated with PH targeted therapy (sildenafil). Twenty-one patients received long-term oxygen therapy (LTOT).

The mean observation period was 2.74±1.63 years. Twenty-four patients died during the observation period, 11 due to advanced respiratory failure, 9 due to acute exacerbation, 1 due to lung cancer, and 3 due to unknown causes. There were no deaths associated with PH. The univariate Cox regression model (Table 4) demonstrated that %FVC, %DLCO, PaO_2_, MMRC, 6-min walk distance (6MWD), lowest SpO2, MPAP, and PVR had statistically significant impacts on survival. The stepwise multivariate Cox regression model (Table 5) demonstrated that MPAP (HR = 1.293; 95% CI 1.130–1.480; p<0.001) and %FVC (HR = 0.958; 95% CI 0.930–0.986; p = 0.004) had statistically significant impacts on survival. There was no correlation between MPAP and %FVC (r = −0.09; p = 0.447). In addition, there was no association between any autoantibody and clinical outcomes (data not shown).

**Table 4:** Results of the univariate Cox proportional hazard model.

Variables	HR	95%CI	P value
Sex			
Male	1		
Female	0.615	0.253–1.500	0.285
Age, years	1.051	0.991–1.115	0.095
BMI	0.953	0.856–1.061	0.375
Smoking status			
Never	1		
Ever	0.957	0.423–2.162	0.915
FVC, %predicted	0.954	0.934–0.974	<0.001
DLCO, %predicted	0.940	0.910–0.971	<0.001
PaO_2_, mmHg	0.946	0.909–0.985	0.007
MMRC	2.312	1.500–3.563	<0.001
6MWD, m	0.994	0.991–0.998	0.001
Lowest SpO_2_, %	0.935	0.896–0.935	0.002
MPAP, mmHg	1.158	1.063–1.261	0.001
Cardiac index, lmin^−1^m^−2^	1.027	0.583–1.808	0.927
PAWP, mmHg	1.010	0.892–1.143	0.873
PVR, Wood units	2.012	1.458–2.777	<0.001

HR: hazard ratio; CI: confidence interval.

**Table 5:** Results of stepwise multivariate Cox proportional hazard model.

variables	HR	95%CI	P value
MPAP, mmHg	1.293	1.130–1.480	<0.001
FVC, %predicted	0.958	0.930–0.986	0.004

Used for variables that were significant in univariate analysis (Table 4).

FVC, DLCO, PaO_2_, 6MWD, Lowest SpO_2_, MPAP.

Table 6 shows the baseline characteristics and physiology of patients using a cutoff point of 20 mm Hg of MPAP. %DLCO, PaO_2_, 6MWD, and lowest SpO_2_ were significantly lower in those with MPAP≥20 mm Hg. MMRC, PAWP, and PVR were significantly higher in those with MPAP≥20 mm Hg.

**Table 6:** Baseline characteristics and physiology of patients with and without PH.

Variables	Non PH MPAP<20 mmHg (N = 82)	PH MPAP≥20 mmHg (N = 18)	P value
Sex(M/F)	51/31	13/5	0.425
Age, years	64.5±8.3	67.9±6.4	0.104
BMI	23.5±3.8	24.8±4.6	0.195
Smoking status			
Ever/never	48/34	13/5	0.283
FVC, %predicted	82.9±19.9	73.2±20.9	0.066
DLCO, %predicted	58.4±19.6	46.8±13.7	0.031
PaO_2_, mmHg	82.0±11.6	75.2±10.6	0.026
MMRC	0.9±0.8	1.7±1.4	0.030
6MWD, m	586.3±121.1	433.4±164.3	0.001
Lowest SpO_2_, %	85.2±8.8	81.5±7.7	0.044
MPAP, mmHg	14.7±3.0	23.0±3.3	<0.001
Cardiac index, l⋅min^−1^⋅m^−2^	3.07±0.74	3.20±0.67	0.351
PAWP, mmHg	6.6±3.2	9.7±2.9	<0.001
PVR, Wood units	1.58±0.75	2.71±1.25	<0.001

Data are presented as means ± SD or numbers.

N = 100 except for FVC (n = 99), DLCO (n = 96), MMRC (n = 95), 6MWD (n = 91), Lowest SpO2 (n = 91).

**Figure 3:**
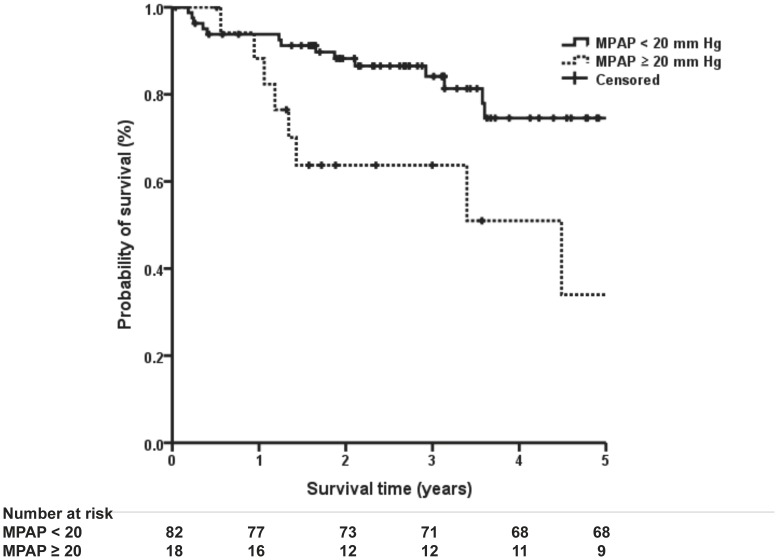
Kaplan-Meier curves for survival according to MPAP (p = 0.005). Survival curves were compared with log-rank statistics.

Figure 3 shows a Kaplan-Meier curve that reveals significantly worse survival among patients whose MPAP was ≥20 mm Hg than among those whose MPAP was <20 mm Hg (log-rank test p = 0.005). The 5-year survival rates from the initial diagnosis were 50.0 and 82.0%, respectively.

## Discussion

This is the first study, to our knowledge, to report the impact of MPAP on survival in LD-CTD patients. This study included patients with milder pulmonary function impairment (mean FVC 81.1%, mean DLCO 56.4%) and milder MPAP **(**mean MPAP 16.2 mm Hg) than subjects of previous studies, however, a higher MPAP was an independent prognostic predictor [2], [12], [27]. This result shows the importance of evaluating MPAP at an early time in patients with LD-CTD.

Using the methods of Kaplan-Meier and the log-rank test, patients with MPAP≥20 mm Hg had a significantly lower survival rate than those with MPAP<20 mm Hg in LD-CTD. Kimura et al. previously demonstrated the importance of PH in IPF patients at their initial workup using a cutoff point (MPAP>20 mm Hg) in RHC [2]. Our studies also demonstrated that borderline PH was found to be closely associated with prognosis in LD-CTD, the same as in IPF. The strength of our study was that RHC was performed on all patients within one month of initial evaluation.

The pathogenesis of PH is likely complex and driven by multiple mechanisms [28]–[29]. As shown in Table 6, patients with PH (MPAP≥20 mm Hg) were found to have a lower PaO_2_ and lowest SpO_2_ during the 6MWT. Previous studies showed that hypoxia induces vasoconstriction and vascular remodeling through various factors, such as vascular endothelial growth factor and hypoxia-inducible factor 1 alpha [30]. Our results indicate that hypoxia may play a crucial role in the development of PH in LD-CTD. Previous studies also suggest a role for autoimmunity in the development of the pulmonary vascular changes in patients with CTD [28]. Therefore, immune and inflammatory mechanisms may also play a significant role in the pathogenesis of PH in patients with LD-CTD. Further investigation will be required to determine whether this is the case.

Another important point of this study is that LD-CTD has several prognostic factors. The univariate Cox regression model demonstrated that %FVC, %DLCO, PaO_2_, MMRC, 6MWD, lowest SpO2, MPAP, and PVR had statistically significant impacts on survival. With stepwise, multivariate Cox proportional analysis, MPAP and %FVC were shown to be independent determinants of survival. Alhamad et al. [12] showed that a lower resting SpO2 level and lower serum albumin were associated with an increased risk of death in LD-CTD, using a univariate Cox model. However, their study did not identify independent predictors of mortality due to the small sample size and low number of deaths. Therefore, this study is the first to identify independent prognostic factors in LD-CTD.

CTD-ILD patients tend to be treated with immunosuppressive therapies, although there have been few systematic and prospective studies [31]–[32]. In addition, a previous study suggested that a minority of CTD-PH patients could also benefit from immunosuppressive therapies [33]. LD-CTD is a new term to describe a subset of ILD patients with features of CTD. However, it is unknown whether the therapeutic strategies and responses of immunosuppressive therapies are the same in LD-CTD as they are in CTD-ILD. In this study, thirty-nine patients were treated with corticosteroid and an immunosuppressive agent. Further trials of therapy are needed for LD-CTD, and may lead to characterization of the subgroup of patients who can benefit from specific therapies.

Our study has several limitations. First, this is a retrospective observational study. Collection of additional prospective data is warranted to confirm our findings. Second, LD-CTD was diagnosed on the basis of the proposed criteria without relying on the suggested histopathological features of CTD [11]. However, the central aim of this study was to investigate the prognostic ability of MPAP and other variables in patients with LD-CTD, and the clinical and physiologic evidence. Since not all patients underwent surgical lung biopsy, exclusion of these patients from the study would introduce significant selection bias. Therefore, we included all patients with LD-CTD who were diagnosed on the basis of serological criteria. Finally, our results are subject to treatment bias. Indications for therapy and choice of drug after the initial evaluation were not uniform among patients, limiting the evaluation of the prognosis.

In conclusion, we demonstrated that %FVC and MPAP have a statistically significant impact on survival in LD-CTD patients. MPAP evaluation provides additional information of disease status and will help physicians to predict mortality in LD-CTD.
